# Amylin Receptor: A Common Pathophysiological Target in Alzheimer’s Disease and Diabetes Mellitus

**DOI:** 10.3389/fnagi.2013.00042

**Published:** 2013-08-15

**Authors:** Wen Fu, Aarti Patel, Jack H. Jhamandas

**Affiliations:** ^1^Division of Neurology, Department of Medicine, Centre for Neuroscience, University of Alberta, Edmonton, AB, Canada

**Keywords:** amyloid-beta protein, amylin, amylin receptor, Alzheimer’s disease, type-2 diabetes mellitus

## Abstract

Amylin (islet amyloid polypeptide) and amyloid-beta (Aβ) protein, which are deposited within pancreatic islets of diabetics and brains of Alzheimer’s patients respectively, share many biophysical and physiological properties. Emerging evidence indicates that the amylin receptor is a putative target receptor for the actions of human amylin and Aβ in the brain. The amylin receptor consists of the calcitonin receptor dimerized with a receptor activity-modifying protein and is widely distributed within central nervous system. Both amylin and Aβ directly activate this G protein-coupled receptor and trigger multiple common intracellular signal transduction pathways that can culminate in apoptotic cell death. Moreover, amylin receptor antagonists can block both the biological and neurotoxic effects of human amylin and Aβ. Amylin receptors thus appear to be involved in the pathophysiology of Alzheimer’s disease and diabetes, and could serve as a molecular link between the two conditions that are associated epidemiologically.

## Introduction

Both Alzheimer’s disease (AD) and type-2 diabetes mellitus (T2DM) are chronic, age-related diseases that share common clinical and biochemical features (Selkoe, 1997; Hoppener et al., 2000; Götz et al., 2009; Wolfson et al., 2009; Tacutu et al., 2010). In addition to epidemiological links between the two diseases (Luchsinger, 2010), insulin-resistant T2DM has been identified as a risk factor for AD (Craft, 2007). Dysregulation of insulin and IGF-1 (insulin growth factor-1) receptors has been observed in AD patient brains (Moloney et al., 2010). Impairment of these receptors contributes to increased amyloid-beta (Aβ) protein aggregation, subsequent synaptic loss, and cognitive impairment (Zhao et al., 2009). T2DM and AD are pathologically characterized by presence of insoluble protein aggregates resulting in formation and deposition of amylin in T2DM and Aβ in AD respectively (Westermark et al., 2011; Abedini and Schmidt, 2013). Amylin or islet amyloid polypeptide (IAPP) is a 37-amino acid peptide first isolated from amyloid-rich pancreatic extracts of type-2 diabetic patients (Cooper et al., 1987). This misfolded protein forms elongated fibrils with spines consisting of many-stranded β sheets and enters the amyloid state (Eisenberg and Jucker, 2012). Amylin is also deposited in human brains in T2DM with dementia and AD patients (Jackson et al., 2013). Both amylin and Aβ directly activate amylin receptors, and the biological effects of these two peptides and their neurotoxic actions can be blocked with specific amylin receptor antagonists (Jhamandas and MacTavish, 2004; Jhamandas et al., 2011). These observations have raised further questions concerning the relationship, at a molecular level, between these two different common clinical conditions and the role that the amylin receptor may play as a common pathological link.

## Amylin Receptors

Amylin receptors consist of heterodimerized complexes of the calcitonin receptor (CTR) interacting with one of receptor activity-modifying proteins (RAMPs). CTR is a seven transmembrane-domain Class B G protein-coupled receptor. The RAMP is a single-domain protein, and not a receptor itself, with three currently known subtypes named RAMP_1–3_. These RAMPs can heterodimerize with CTR to form a subset of amylin receptors (AMY_1–3_) or in combination with calcitonin receptor-like receptor (CLR) form either calcitonin gene-related peptide (CGRP) or adrenomedullin receptors (Hay and Poyner, 2013). The physiological roles ascribed to amylin are mediated via the amylin receptors (Muff et al., 1999). Distribution of amylin and its receptors is fairly widespread in the brain. The most investigated of amylin functions in the brain relate to control of energy homeostasis and body fluid balance through amylin receptors (Lutz, 2012). In rat, the amylin neural circuit includes the area postrema, a structure devoid of the blood brain barrier, the nucleus of the solitary tract, the lateral parabrachial nucleus, and the central nucleus of the amygdala (Roth et al., 2009). In postpartum rats, amylin levels are significantly elevated in the preoptic nuclei suggesting that this peptide is also involved in maternal physiological functions (Dobolyi, 2009). However, its role in cognitive function is incompletely understood in spite of the localization of this peptide in many areas related to memory and learning. At present, there is no information available on age-related changes in the expression of amylin or its receptors in the brain.

## Aβ and Alzheimer’s Disease

The amyloid plaque is one of core pathological hallmarks in AD (Hardy, 2006; Ballard et al., 2011). Aβ is a 40- or 42-amino acid peptide cleaved from a larger precursor, amyloid precursor protein (APP), by the actions of β- and γ-secretases. Aβ can form monomers, oligomers, or fibrils. Small soluble oligomers of Aβ are deemed to be more toxic than mature fibrils (Ballard et al., 2011). AD is a complex neurodegenerative disease caused by multiple dysregulated neurobiological networks and cellular functions, neuronal, and memory loss. It is clear that soluble oligomeric Aβ plays a key role in the pathogenesis of AD, although as a therapeutic target in this condition, it has posed significant challenges, and as yet, is of unproven benefit.

## Amylin and Type-2 Diabetes

Amylin is co-secreted with insulin by pancreatic beta-cells but is greatly decreased in diabetic individuals following food intake. It has the propensity to form membrane permeant toxic oligomers and aggregate into fibrils in much the same way as Aβ. These toxic amylin oligomers contribute to pancreatic beta-cell loss in T2DM (Haataja et al., 2008). The effects of amylin on glucose metabolism and food intake are mediated by the peripheral and central mechanisms (Young, 2005). Amylin concentration is only 1–2% that of insulin and concurrently increases with insulin after glucose stimulation. It inhibits glucagon release and influences insulin secretion in a concentration-dependent manner (Akesson et al., 2003). Amylin also inhibits gastric emptying in a manner similar to glucagon-like peptide-1 (GLP1) and decreases food intake via central autonomic mechanisms (Rushing et al., 2000). Although many of amylin functions are still not fully understood, there are two plausible physiological roles of amylin that are of particular interest. The first relates to its function as an auto- or paracrine molecule in the islets of Langerhans, and the other is its role as a neurohormone with effects on the central nervous system (CNS) (Westermark et al., 2011). The amylin fibrillogenicity is species dependent. Although there is only a six amino acid difference between human and rat amylin, human amylin, but not rat, is amyloidogenic and demonstrates neurotoxic properties (Lim et al., 2008).

## Amylin and Aβ

Although there is no sequence homology between Aβ and human amylin, both aggregate and form amyloid fibrils. In this process, they share the same important features which include sequence-specific aggregation with oligomeric intermediates as precursors to the aggregated state and the physical state of inclusion bodies. The amyloid fibrils are typically unbranched, variable in length, polymorphic, and form crossed β-sheet structures (Mitraki, 2010; Abedini and Schmidt, 2013). The amyloid plaque formation could be a dynamic process between protein monomers, oligomers, and fibrils. Amyloid fibrils can be an integral part of normal cellular physiology and serve as storage reservoirs for peptide hormones within secretory granules (Maji et al., 2009). Although Aβ or amylin insoluble fibrils may play some physiological/pathophysiological roles, it is the soluble oligomeric intermediates that are believed to represent the primary toxic species of amyloids (Hardy and Selkoe, 2002). Many soluble oligomers from different proteins (including Aβ, amylin, prion protein, alpha-synuclein) display a common conformation-dependent structure that is unique to soluble oligomers regardless of sequence. The oligomer toxicity is inhibited by oligomer-specific antibodies, which suggests that different types of soluble amyloid oligomers have a common structure and share a common mechanism of toxicity (Kayed et al., 2003). Interestingly, human amylin shows strikingly similar neurotoxicity profiles with Aβ (May et al., 1993; Lim et al., 2008, 2010). Amylin receptor antagonists, AC187 or AC253, attenuate or block Aβ- and amylin-induced neurotoxicity, potassium channel activity, and activation of pro-apoptotic genes (Jhamandas et al., 2003, 2011; Jhamandas and MacTavish, 2004). Downregulation of amylin receptor gene expression using siRNA attenuates the oligomerized Aβ-induced toxicity (Jhamandas et al., 2011). Moreover, in AD transgenic model mice (TgCRND8) which over-express APP, amylin receptor expression in the brain was up-regulated in an age-dependent manner within specific brain regions that also demonstrated an increased amyloid burden (Jhamandas et al., 2011). Aβ, in a manner identical to human amylin, can directly activate AMY3 to raise cyclic adenosine monophosphate (cAMP), increase intracellular calcium, and PKA and MAPK phosphorylation (Fu et al., 2012). Furthermore, AC253 acting via AMY3 receptors, can re-establish the long-term potentiation (LTP) in hippocampus of AD mice (TgCRND8) (Kimura et al., 2012). Thus, many of the effects of Aβ, at a cellular level, appear to be expressed via the amylin receptor and such observations support the presence of a direct interaction between oligomer Aβ and amylin receptors.

## Insulin, IDE, IGF-1 and 2, and Insulin Receptors in Diabetes and Alzheimer’s Disease

Recently, based on evidence of shared pathophysiology between AD and T2DM, AD has been proposed to represent “Type 3 diabetes” (Steen et al., 2005). Several candidate proteins have been proposed to bridge the pathophysiological link between the two conditions. The major mechanism through which T2DM may influence AD includes central insulin resistance, which leads to reduced sensitivity to insulin in the brain, resulting in hyperinsulinemia, impaired insulin receptor (IR) signaling, and glucose toxicity (Freude et al., 2009; Han and Li, 2010). T2DM mediated hyperinsulinemic/hypoglycemic episodes may produce long-term changes in brain vasculature, cellular toxicity including inflammation and oxidative stress, alternations in Aβ levels, tau phosphorylation, neurodegeneration, and cognitive impairment, thus facilitating AD onset. It is clear that insulin and IGF-1 have intense effects in the CNS, acting as neuromodulators to influence release and uptake of neurotransmitters, energy homeostasis, neuronal survival, as well as learning and memory (Bosco et al., 2011). There is a decrease in insulin mRNA and protein levels (Steen et al., 2005) and a marked disturbance of IR and IGF-1R signaling in the CNS of AD patients (Frölich et al., 1998). Post-mortem examination of brains from patients with AD revealed substantial downregulation of IR, IGF-1R, and insulin receptor substrate (IRS) proteins (Squire, 1986), that correlate with the severity and progression of neurodegenerative changes in this condition (Frölich et al., 1998). Insulin resistance has been postulated to interfere with Aβ catabolism and clearance in AD pathogenesis (Qiu and Folstein, 2006). Impairment in IGF-1 is linked to Aβ pathology and IGF-1 increases Aβ clearance from brain in AD animal models (Carro et al., 2006). IDE is the chief enzyme that degrades excess insulin and other substrates, including Aβ (Farris et al., 2003). The imbalance of the substrates could affect the IDE-induced degradation process, and in the process influence the pathogenesis of AD or T2DM. Increasing the amount of insulin appears to both stimulate Aβ secretion and also inhibit the IDE enzymatic degradation of extracellular Aβ, thus resulting in increased Aβ neurotoxicity (Qiu and Folstein, 2006). Additionally, IDE knockout mice experience decreased Aβ degradation, hyperinsulinemia, and hyperglycemia (Farris et al., 2003).

**Figure 1:**
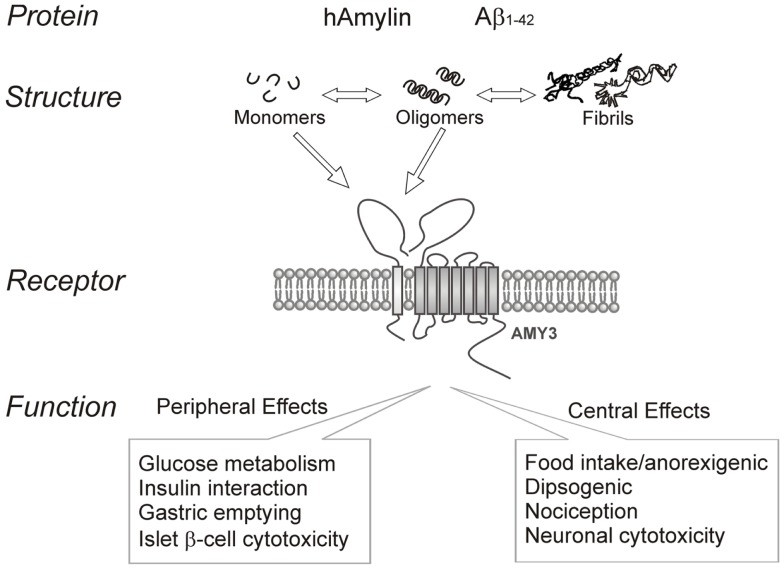
**Summary of human amylin and amyloid-beta (Aβ) interactions with the amylin receptor (AMY3) and functional consequences of such interactions at the peripheral and central levels**.

In summary, amyloid formation is a fundamental process observed in many protein misfolding diseases including AD and T2DM. Increasing evidence indicates that the intermediate oligomers from different proteins form common conformation-dependent structures that play primary pathological roles in neurodegenerative conditions. One common target for the soluble oligomers Aβ and human amylin is the amylin receptor. Figure 1 summarizes our perspective on the commonality of structure-functional relationships between the two proteins. Activation of the amylin receptor by the two peptides can modulate activity of individual neurons at a cellular and synaptic level, but more chronic exposure to these peptides results in activation of signal transduction pathways that culminate in apoptotic cell death. Currently, the treatment of AD remains a serious and important public health concern. Beyond symptomatic treatment, there is as yet no effective intervention for AD. Targeting amylin receptors using amylin receptor antagonists to block the deleterious effects of Aβ and human amylin could represent a novel therapeutic approach to treating AD and T2DM, conditions that are linked epidemiologically and apparently also at a molecular level.

## Conflict of Interest Statement

The authors declare that the research was conducted in the absence of any commercial or financial relationships that could be construed as a potential conflict of interest.
